# Gene Therapy for Neurodegenerative Diseases: Slowing Down the Ticking Clock

**DOI:** 10.3389/fnins.2020.580179

**Published:** 2020-09-18

**Authors:** Raygene Martier, Pavlina Konstantinova

**Affiliations:** Department of Research and Development, uniQure Biopharma B.V., Amsterdam, Netherlands

**Keywords:** gene therapy, neurodegenarative disease, ALS, AAV (adeno-associated virus), RNA interference (RNAi)

## Abstract

Gene therapy is an emerging and powerful therapeutic tool to deliver functional genetic material to cells in order to correct a defective gene. During the past decades, several studies have demonstrated the potential of AAV-based gene therapies for the treatment of neurodegenerative diseases. While some clinical studies have failed to demonstrate therapeutic efficacy, the use of AAV as a delivery tool has demonstrated to be safe. Here, we discuss the past, current and future perspectives of gene therapies for neurodegenerative diseases. We also discuss the current advances on the newly emerging RNAi-based gene therapies which has been widely studied in preclinical model and recently also made it to the clinic.

## AAV Gene Therapy

Gene therapy is an emerging therapeutic tool used to deliver functional genetic material to cells in order to correct a defective gene. By delivering a copy of a therapeutic gene to affected cells, the product encoded by that gene [i.e., its messenger RNA (mRNA) and/or proteins] will be continuously synthesized within the cell, utilizing the cell’s own transcriptional and translational machinery (Porada et al., 2013). The main advantage of this technology is that it offers a potentially life-long therapeutic effect without the need for repeated administration. Gene therapy can be used to correct defective genes by introducing a functional copy of the gene, by silencing a mutant allele using RNA interference (RNAi), by introducing a disease-modifying gene, or by using gene-editing technology (Grimm and Kay, 2007; Dow et al., 2015; Saraiva et al., 2016).

Gene therapy vectors can be either viral or non-viral. Different physical and chemical systems can be applied to deliver therapeutic genes to cells without the need of a viral vector. Non-viral vectors have no size limitation for the therapeutic gene, generally have a low immunogenicity risk, and can be produced at relatively low costs (Nayerossadat et al., 2012). However, due to the fact that high therapeutic doses are required when using non-viral technologies, and the resulting gene expression is generally transient, most gene therapies now rely on viral vectors. Numerous viral vector types have been tested in clinic, including vaccinia, measles, vesicular stomatitis virus (VSV), polio, reovirus, adenovirus, lentivirus, γ-retrovirus, herpes simplex virus (HSV) and adeno-associated virus (AAV) (Lundstrom, 2018). Vaccinia, measles, VSV, polio, reovirus, adeno, and HSV vectors are currently mainly used in either vaccines or cancer therapeutics, while lentiviral and γ-retroviral vectors are predominantly used for transduction of transplantable cells (Finer and Glorioso, 2017). For *in vivo* gene delivery, AAV is currently the preferred vector.

**FIGURE 1:**
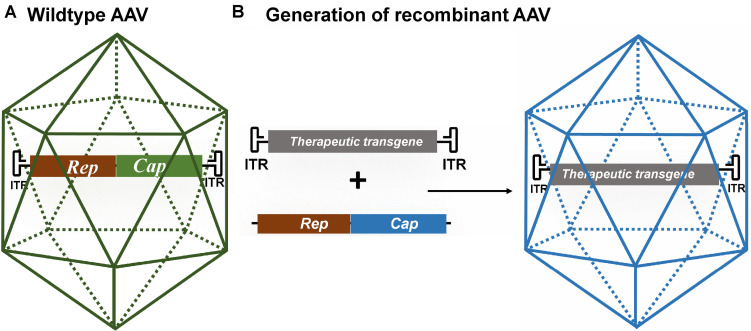
**(A)** Schematic of wild-type AAV. Its genome consists of the viral Rep and Cap genes flanked by two inverted terminal repeats (ITRs). **(B)** Schematic of recombinant AAV (rAAV) generated by replacing the viral genes for a therapeutic gene. The therapeutic transgene expression cassette consists of a promoter of choice, a therapeutic gene and a polyadenylation signal (not shown) and is flanked by the AAV ITRs. The Rep and Cap genes are expressed from a different plasmid or viral vector. The rAAV is generated by co-transfecting cells with the transgene cassette flanked by AAV ITRs, the Rep and Cap genes of a specific AAV serotype, and an adenovirus helper plasmid.

Adeno-associated virus belongs to the Parvoviridae family and is preferred for gene therapy because it is non-replicating (AAV requires a helper virus for replication), has a low immunogenicity profile, and is not known to cause disease (Aschauer et al., 2013; Naso et al., 2017). In the absence of a helper virus, AAV may stably integrate into the host genome, but at a relatively low frequency. The genome of wild-type AAV is about 4.7 kb and is flanked between two inverted terminal repeats (ITRs) (Figure 1A) (Ojala et al., 2015). The open reading frame between the ITRs contains a replication (Rep) gene and a capsid (Cap) gene. The ITRs are *cis-*acting elements and are required for genome replication, integration, and packaging into the capsid. The Rep gene encodes four proteins (Rep78, Rep68, Rep52, and Rep40) that have important roles in replication and encapsidation of the viral DNA (Collaco et al., 2003). The Cap gene encodes three capsid proteins (VP1, VP2, and VP3) and an assembly activating protein that promotes capsid formation (Sonntag et al., 2010). There is a growing number of naturally occurring and engineered AAV serotypes with different viral capsids that have altered tissue tropism, transduction rate, or other features such as the ability to cross the blood–brain barrier (Zincarelli et al., 2008; Deverman et al., 2016). Recombinant AAV (rAAV) can be produced by replacing the Rep and Cap genes with an expression cassette containing a therapeutic gene of interest (Figure 1B). Formation of wild-type AAV is prevented by expressing the Rep and Cap genes on a separate plasmid (AAV packaging plasmid or AAV helper plasmid). The ITRs are the minimal regions required to be retained in rAAV to allow packaging of its genome. rAAV might still integrate randomly in the human genome at very low frequencies but most of its genome is maintained as episomal circular structures known as concatamers. rAAVs have been widely used in over 200 human clinical studies and they have shown to be safe (Hudry and Vandenberghe, 2019). AAV-based gene therapies are highly attractive for the treatment of neurodegenerative diseases due to the neuronal tropism and their good safety profile demonstrated in clinical studies. In addition, a single administration results in long-term, potentially life-long, gene expression which is a main advantage when *in vivo* AAV administration requires invasive procedures.

## Neurodegenerative Diseases

Neurodegenerative diseases are a heterogeneous group of multi-system disorders affecting the central nervous system, ultimately leading to neurodegeneration (Gitler et al., 2017). Examples of the most common neurodegenerative diseases are amyotrophic lateral sclerosis (ALS), frontotemporal dementia (FTD), spinocerebellar ataxias (SCAs), Huntington’s disease (HD), Alzheimer’s disease (AD), and Parkinson’s disease (PD) (Bertram and Tanzi, 2005). The prevalence of these largely age-dependent disorders is increasing, partly due to the aging population, which in turn places a major economic burden on health care services. Some neurodegenerative diseases are caused by genetic mutations and/or cellular and circuit dysregulation. In some cases, different neurodegenerative diseases are linked to the same polymorphisms or mutations, thereby sharing similar pathological mechanisms. It seems that certain environmental or lifestyle factors combined with genetic factors increase the risk for certain neurodegenerative disease but aging is considered to be the most important risk factor for the sporadic cases (Brown et al., 2005). Although each neurodegenerative disease has a different pathophysiology, they all lead to damage to the nervous system due to features such as cell death, impaired/failure of axonal regeneration, demyelination, and/or structural and functional neuronal deficits (Hussain et al., 2018). These pathological features occur in different combinations and their causes can be either genetic or unknown (Hussain et al., 2018). Common underlying causes leading to these conditions can be attributed to abnormal accumulation of proteins such as amyloid in AD, misfolded proteins (typical for PolyQ diseases), aggregation of proteins such as Tau (AD and traumatic brain injuries), synuclein (PD) or TDP-43 (ALS), RNA toxicity, or translational products from repeats expansion within genes (Blokhuis et al., 2013; Hussain et al., 2018). Each of these features has unique mechanisms of toxicity which in large part are currently not well-understood. Some mechanisms in pathogenesis of neurodegenerative diseases share key characteristics with prions suggesting that progression of certain neurodegenerative diseases may share similar mechanisms involved in prion diseases (Frost and Diamond, 2010). For example, misfolded protein aggregates (such as Tau, α-synuclein, amyloid-ß, huntingtin) can spread via cell to cell interaction and invade healthy tissues. The misfolded proteins can induce secondary misfolding of other unrelated aggregation-prone proteins, impairing the entire proteostatic network (Sweeney et al., 2017). Ultimately, they lead to a decline or even the complete loss of sensory, motor, and cognitive functions. Symptoms commonly associated with neurodegenerative diseases include cognitive impairment, memory loss, apathy, anxiety, muscle weakness, paralyzes, and difficulties with speech or breathing.

One great mystery for most neurodegenerative diseases is the onset of clinically manifest symptoms, as pathological alterations usually occur long before symptoms start to develop. Progressive accumulation of neuronal cell damage and the effects of aging are two common explanations, and more recently, the role of neuroplasticity in the development and progression of neurodegeneration has also been implicated (Schaefers and Teuchert-Noodt, 2016). The adult brain generally shows less neuroplasticity in response to insults than the developing nervous system (Schaefers and Teuchert-Noodt, 2016). However early-life events and insults such as perinatal infections, an unstructured/abusive environment, social isolation, stress, poor nutrition, exposure to chemicals or metals could possibly interfere with the neuroplastic development in children and adolescents (Logroscino, 2005; Mortimer and Borenstein, 2007; Miller and O’Callaghan, 2008). These may place an additional burden on the plastic capacity of the developing neuronal system, leading to the disturbance of the structural brain self-organization. A second challenge later in life could trigger the final onset of neurodegeneration and this may be a critical factor in determining the onset and course of neurodegeneration (Schaefers and Teuchert-Noodt, 2016).

A major clinical challenge is the early diagnosis of neurodegenerative diseases, and due to overlapping symptoms discrimination between the different diseases is difficult. Moreover, early symptoms are often dismissed or interpreted as normal consequences of aging. Apart from diagnostic delay, additional challenges for new therapeutic approaches reaching the stage of clinical development include the lack of druggable targets, the limited choice of delivery methods, and a lack of reliable biomarkers and clinical parameters that predict therapeutic efficacy or the rate of disease progression. To date, neurodegenerative diseases cannot be cured, and only palliative treatments are available. As these diseases are devastating for patients and their families, in turn also causing a vast burden on society, there is an enormous need to better understand their causes, pathology, and clinical progression, and to develop early detection methods and new therapeutic interventions.

In this review we will provide an up-to-date overview of the development and characterization of gene therapies for neurodegenerative diseases with focus on two common neurodegenerative diseases, ALS and SCA3, each with a different pathology. In addition, we will outline the clinical studies in neurodegenerative diseases using adeno-associated viral vector (AAV) and will address some of the major translational challenges in this emerging therapeutic field.

## Amyotrophic Lateral Sclerosis

ALS (or Lou Gehrig’s disease) is the most common adult onset motor neuron disease affecting the upper- and the lower motor neurons in the brain and spinal cord, but other neuroanatomical regions may also be affected (Geser et al., 2010; Oskarsson et al., 2018). The upper motor neurons are found in the motor cortex of the brain, while the lower motor neurons are located along the brainstem, spinal cord and extend to the muscles (Ravits and La Spada, 2009). Degeneration of the upper motor neurons causes symptoms such as spasticity and hyperreflexia while the loss of lower motor neurons results in progressive muscle weakness, cramps, fasciculations, muscle wasting, and paralysis (Ravits and La Spada, 2009; Ferrari et al., 2011). The prevalence of ALS is currently estimated at 5 in 100.000 but the estimated lifetime risk to develop the disease is about 1:400–800 (Bennion Callister and Pickering-Brown, 2014; Ingre et al., 2015). The discordance between the low prevalence but high lifetime risk is explained by the fact that ALS patients have a very limited life span with a median survival ranging from 2 to 5 years from symptom onset (Oskarsson et al., 2018). The understanding of the genetic causes of ALS is continually expanding, but our knowledge of other risk factors such as environmental factors, lifestyle, or aging remains poor. Only 10% of ALS cases are familial, and the causal genetic mutations are usually inherited in a mendelian autosomal dominant manner (Siddique and Ajroud-Driss, 2011). Thus, most ALS cases are assumed to occur sporadically, and the main causes are still unknown.

**FIGURE 2:**
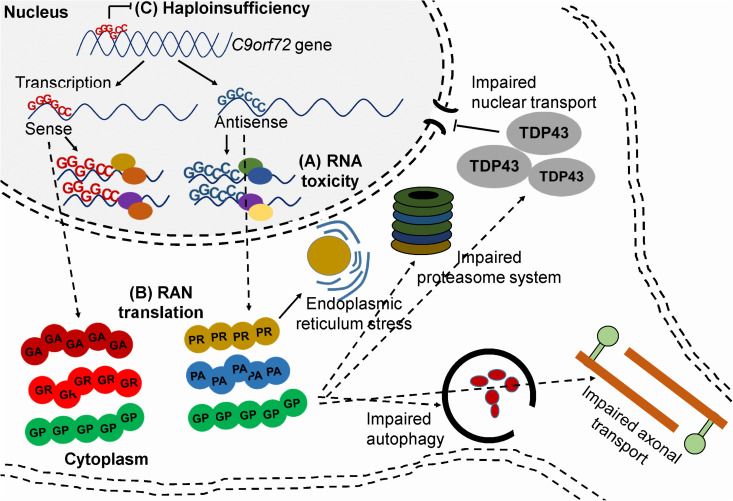
Mechanisms of toxicity associated with *C9orf72* G_4_C_2_ repeat. **(A)** RNA-mediated toxicity. Repeat-containing sense and antisense RNA transcripts accumulate and sequester RNA binding proteins **(B)** RAN translation. The sense and antisense repeat-containing transcripts undergo RAN translation into five, potentially toxic DPRs **(C)** Haploinsufficiency. Hypermethylation of the expansion leads to reduced transcription of *C9orf72*.

Two drugs have been approved by the Food and Drug Administration for the treatment of ALS, but the efficacy of both drugs is modest. Riluzole – first approved in 1995 – is a glutamate receptor antagonist which may increase survival by 2 to 3 months. More than two decades later in 2017, Edaravone, a free radical scavenger was approved but its efficacy is still unclear, and at best the drug has a moderate effect on disease progression (Cruz, 2018). More than 30 genes have been linked to ALS and mutations in chromosome 9 open reading frame 72 (*C9orf72)*, superoxide dismutase1 (SOD1), transactive response DNA-binding protein 43 (TDP-43), or fused in sarcoma (FUS) are responsible for most of the familial ALS cases. A hexanucleotide expansion consisting of GGGGCC (G_4_C_2_) nucleotides in the first intron of the *C9orf72* gene is the most frequent genetic cause of ALS and is found in about 40–50% of familial ALS cases and 5–10% of sporadic ALS cases (Umoh et al., 2016). The G_4_C_2_ repeat is transcribed bidirectionally and affected patients usually carry more than 30 G_4_C_2_ copies. Interestingly, the same mutation also causes FTD, the second most common form of dementia after AD. *C9orf72* is responsible for 25% of familial FTD cases and 5–7% of sporadic FTD cases. ALS and FTD are considered overlapping diseases as about 15% of ALS patients develop FTD and up to 50% of ALS patients show some degree of functional loss in the frontal lobe of the brain (Bennion Callister and Pickering-Brown, 2014). The function of the *C9orf72* encoded protein is poorly understood, but it may be a regulator of the autophagy-lysosome pathway during nutrient stress responses (Liu and Wang, 2019). There are at least three proposed pathogenic mechanisms in *C9orf72* related ALS and/or FTD patients; (1) RNA-mediated toxicity, (2) repeat-associated non-ATG (RAN) translation, (3) haploinsufficiency. It is also possible that a combination of the three mechanisms contributes to the disease pathogenesis (Figure 2) (Kumar et al., 2017; Shi et al., 2018).

### RNA-Mediated Toxicity

RNA-mediated toxicity was first described in myotonic dystrophy type 1 (DM1), which is caused by a CTG repeat expansion in the 3′ UTR of the myotonic dystrophy protein kinase gene (Mahadevan et al., 1992; Taneja et al., 1995). It was shown that RNA consisting of CUG repeats folds into stable structures that colocalize with RNA-binding proteins and sequester their function. For example, an important protein that is sequestered by CUG-containing RNA foci is the splicing factor muscleblind-like 1 (MBNL1) (Mankodi et al., 2000). The sequestration of MBNL1 leads to its inactivation which subsequently causes mis-splicing of several pre-mRNAs, such as muscle-specific chloride ion channels and insulin receptors (Mankodi et al., 2002; Dansithong et al., 2005). RNA foci are observed in several other neurodegenerative diseases caused by repeat expansions including ALS and SCA3 (Belzil et al., 2013). In *C9orf72*-related ALS and FTD, accumulation of sense and antisense G_4_C_2_-containing RNA foci is detected in several brain-, spinal cord tissues, lymphocytes and fibroblasts. RNA foci are also detected in patient-derived induced-pluripotent stem cell (iPSC)-neurons. Although RNA foci mainly accumulate in the nucleus of cells they are also observed at lower concentrations in the cytoplasm. Several RNA binding proteins interact with G_4_C_2_ RNA repeats, such as ADARB2, hnRNPA1, hnRNPA1B2, Pur-α, FUS, Nucleolin, and TDP-43 (Donnelly et al., 2013; Sareen et al., 2013; Kumar et al., 2017). However, the contribution of these proteins to neurodegeneration is only partially understood.

### RAN Translation

Another proposed mechanism of toxicity in *C9orf72* related ALS and/or FTD is through RAN translation (Stepto et al., 2014; Peters et al., 2015; Kumar et al., 2017). It has been shown that the repeat-containing transcripts can be translated into dipeptide repeat proteins (DPRs) even in the absence of an ATG start codon and even though the mutation is in a non-coding region of *C9orf72*. These DPR proteins are toxic and they can form aggregates that accumulate in the brain and spinal cord of patients (Zu et al., 2011; Zu et al., 2013). Six DPR proteins can be produced via unconventional translation in all reading frames. Glycine-alanine (GA) and glycine-arginine (GR) are produced from the sense repeat-containing transcripts, while proline-arginine (PR) and proline-alanine (PA) are produced from the antisense repeat-containing transcripts. Glycine-proline (GP) is produced from both sense and antisense repeat-containing transcripts (Gitler and Tsuiji, 2016). Ample evidence indicates that DPR proteins are toxic and cause neurodegeneration. For example, neurotoxicity, proteasome activity, and endoplasmic reticulum stress were observed in primary neurons expressing GA proteins (Zhang et al., 2014). The addition of recombinant GR and PR proteins to Hela cells and human astrocytes was toxic and caused alterations in RNA processing (Gitler and Tsuiji, 2016). Expressing GR and PR proteins in Drosophila caused toxicity and early lethality (Mizielinska et al., 2014). Several recent publications have demonstrated that DPR proteins can lead to the impairment of nuclear transport, causing accumulation of several RNA-binding proteins including TDP-43 in the cytoplasm (Freibaum et al., 2015; Jovičič et al., 2015; Zhang K. et al., 2015). Cytoplasmic TDP-43 aggregation is observed in ∼97% of cases of ALS, including those associated with *C9orf72* mutations. TDP-43 protein is predominantly found in the nucleus but can shuttle between the nucleus and cytoplasm to regulate processes such as RNA processing, transcription, pre-mRNA splicing, transport and stabilization of mRNA (Chen-Plotkin et al., 2010; Warraich et al., 2010; Moujalled et al., 2017). In patients, TDP-43 accumulates as cytosolic inclusions with a C-terminal fragment of 25 or 35 kDa. The aggregated TDP-43 is also post-translationally modified, being heavily ubiquitinated and phosphorylated at the C-terminal region (Moujalled et al., 2017). Notably, mutations in TARDBP, the gene that encodes the TDP-43 protein, have also been linked to ALS, but it remains unclear whether it is depletion of nuclear TDP-43, gain of a toxic function of cytoplasmic TDP-43, or a combination of these processes that are neurotoxic.

Furthermore, it has been recently demonstrated that expressing PR in mice also resulted in neurodegeneration and premature death (Zhang et al., 2019). PR proteins localized to the heterochromatin caused abnormal histone H3 methylation and aberrations in nuclear lamins and heterochromatin protein 1α (HP1α). This resulted in down-regulation of numerous differentially expressed genes and upregulation of many repetitive elements accompanied by the accumulation of double-stranded RNA, ultimately leading to neuronal death (Zhang et al., 2019). Thus, as RNA foci and DPRs both contribute to the pathogenesis of ALS, their inhibition could potentially reduce the disease burden in patients.

### Haploinsufficiency

The thought that haploinsufficiency may contribute to *C9orf72*-related ALS originates from the finding that both lower RNA and protein levels have been reported in brain and spinal cord tissues and in iPSC-neurons derived from ALS patients. Several mechanisms may lead to reduced levels of *C9orf72*, including abortive transcription caused by G-quadruplexes and R-loop structures of the repeat-containing transcripts. In addition, the *C9orf72* locus is hypermethylated in several mutation carriers, which can lead to epigenetic silencing. Similar mechanisms of gene silencing have been observed in other repeat expansion diseases such as Friedrich ataxia and fragile X mental retardation syndrome. However, haploinsufficiency alone does not explain the observed ALS pathology in patients. Several loss-of-function mutations in *C9orf72* have been identified and appeared to be non-pathogenic (Harms et al., 2013). Furthermore, complete *C9orf72* knockout mice showed no neurodegeneration but rather splenomegaly, enlarged cervical lymph nodes, and autoimmune related premature death (Koppers et al., 2015; Jiang et al., 2016). Interestingly, this phenotype was rescued in mice hemizygous for *C9orf72*, suggesting that a partial knockdown of the gene would be well-tolerated (Jiang et al., 2016). A recent publication suggests a cooperative pathogenesis between gain- and loss-of function mechanisms (Shi et al., 2018). Reduced *C9orf72* protein in cultured motor neurons caused accumulation of glutamate receptors and excitotoxicity in response to glutamate. In addition, these motor neurons showed impaired clearance of DPRs and were hypersensitive for these proteins.

As most evidence points toward a toxic gain of function resulting from this mutation, current therapeutic strategies are mainly based on silencing of the mutated gene. For example, with antisense oligonucleotides (ASOs) or RNAi (Lagier-Tourenne et al., 2013; Martier et al., 2019a,b).

## Spinocerebellar Ataxia Type 3

The spinocerebellar ataxias (SCAs) are a large group of neurodegenerative diseases that are characterized by progressive ataxia due to degeneration of the cerebellum and often adjacent regions. SCAs are inherited in an autosomal-dominant manner and have a prevalence of about 1–3 in 100.000, although this highly varies based on geography and ethnicity (Rosenberg, 1992; Rüb et al., 2008). More than 40 different types of SCA have been identified and the most common ones (SCA1, SCA2, SCA3, SCA6, SCA7, and SCA17) are caused by a CAG nucleotide repeat expansion encoding polyglutamine (polyQ) (Paulson et al., 2017). SCA3 (also known as Machado-Joseph disease or MJD) is the second most common polyQ disorder after HD and the most common amongst the SCAs. SCA3 patients experience progressive ataxia affecting balance, gait, and speech and frequent symptoms are pyramidal signs, progressive external ophthalmoplegia, dysarthria, dysphagia, rigidity, distal muscle atrophies, and double vision (Evers et al., 2014). The pathogenic CAG repeat is in the penultimate exon of ATXN3 gene on chromosome 14q32.1 and the disease severity is related to the number of CAG repeats (Kawaguchi et al., 1994). Up to 44 CAG repeats are considered normal, between 45 to 51 repeats are associated with intermediate or low penetrance of the disease, while SCA3 patients usually have more than 51 repeats. The survival rate is variable and usually between 10 and 21 years after symptom onset (Kieling et al., 2007). Similar to ALS, there is no cure for SCA3 and current treatments are based on antispasmodic drugs to help reduce spasticity, speech therapy, and physiotherapy. Several molecules such as sulfamethoxazole-trimethoprim, varenicline, and lithium carbonate have been tested in clinical trials, but all failed to show clinical improvement.

The *ATXN3* gene encodes a 42-kDa protein called ataxin-3 which consists of an N-terminal catalytic Josephin domain and two to three (dependent on the splice variant) C-terminal ubiquitin (Ub)-interacting motifs flanking the polyQ tract (Paulson et al., 1997, 2017; Evers et al., 2014). The ataxin-3 protein is widely expressed in different cell types of peripheral and neuronal tissues and present in both nucleus and cytoplasm (Macedo-Ribeiro et al., 2009). Ataxin-3 is believed to interact with up to 100 proteins involved in ubiquitin-dependent pathways and quality control. The protein has a role in various ubiquitin-dependent pathways that maintain protein homeostasis. By partnering with ubiquitin ligases, ataxin-3 may be able to regulate, or edit, the lengths and linkage types of ubiquitin chains on proteins, and in this manner either rescue proteins from being degraded or stimulate protein breakdown (Burnett et al., 2003; Todi et al., 2009). Additional roles in endoplasmic reticulum-associated degradation, aggresome (aggregates of misfolded proteins) production and DNA repair have also been implicated (Wang et al., 2012; Chatterjee et al., 2015; Gao et al., 2015; Ashkenazi et al., 2017; Minoia et al., 2017).

### Mechanisms of Toxicity

**FIGURE 3:**
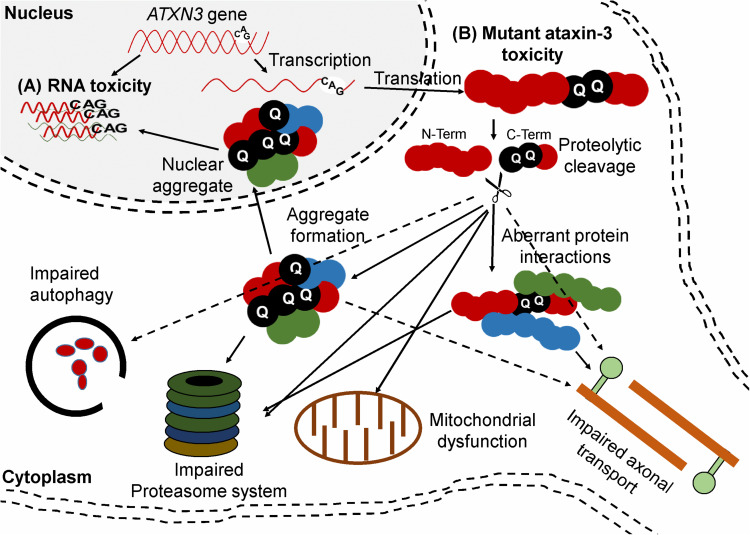
Ataxin-3 mediated mechanisms of toxicity. **(A)** RNA toxicity. RNA containing the CUG repeats can sequester function of transcription factor **(B)** Mutant ataxin-3 toxicity. The CAG repeat-containing ATXN3 transcript is translated into a protein with a polyQ expansion. Proteolytic cleavage of mutant ataxin-3 can generate C-terminal protein fragments containing the polyQ repeat. The mutant ataxin-3 and C-terminal protein fragments can both cause several cellular disturbances such as transcriptional deregulation, impaired autophagy, mitochondrial dysfunction, proteasomal impairment, compromised axonal transport, and DNA damage.

The mutant ataxin-3 protein is neurotoxic but the exact pathways leading to neurodegeneration are not completely understood. Two attractive hypotheses postulate a gain of toxicity and RNA-mediated toxicity (Figure 3). The expanded CAG repeat in the ATXN3 gene leads to formation of an ataxin-3 protein with an expanded polyQ tract at its C-terminal region with toxic gain of function properties. In addition, the mutated protein causes aggregate formation in neurons which is typical for polyQ diseases. Aggregates are found in different types of neurons in the brain stem (ventral pons), substantia nigra, globus pallidus, dorsal medulla, and dentate nucleus (Paulson et al., 1997; Evers et al., 2014). Although the aggregates are mainly detected in the nucleus, they are also found at low levels in cytoplasm of neurons in affected areas and in axons within fiber tracts known to undergo neurodegeneration in the disease (Matos et al., 2018). The aggregates consist of different types of proteins such as ataxin-3 (wild-type and mutant), heat-shock proteins, transcription factors, and other polyQ disease-associated proteins. It is believed that sequestering of these functional proteins contributes to cellular dysfunction and neurodegeneration. Furthermore, proteolytic cleavage of the mutant ataxin-3 may lead to generation of shorter soluble polyQ fragments that are also toxic. Another hypothesis is that the polyQ expansion induces conformational changes in ataxin-3 and alters its function in multiple ubiquitin-dependent pathways that can lead to altered binding properties, loss of protein function, disorganized subcellular localization, aggregation, and altered proteolytic cleavage (Jana and Nukina, 2004; Evers et al., 2014).

In addition to the mechanisms described above, which are based on protein toxicity, RNA toxicity could also contribute to the disease. As mentioned before, RNA containing CUG repeats can sequester various transcription factors and undergo RAN translation. A crucial piece of evidence for RNA toxicity was observed in nematodes. CAG repeats cloned into the 3′ UTR of a marker protein showed severe toxicity in a length-dependent manner in *Caenorhabditis elegans (C. elegans*). The CUG RNAs formed RNA foci and colocalized with *C. elegans* muscleblind protein. The highest CAG repeats were embryonically lethal while the shorter CAG repeats were tolerated (Wang et al., 2011; Nalavade et al., 2013). Since the mutated ATXN3 gene produces a protein with a toxic gain of function, it is considered a promising target for gene silencing approaches such as ASOs and RNAi-based gene therapy (Evers et al., 2014; Toonen et al., 2017; Martier et al., 2019c).

### *In vivo* Models for ALS and SCA3

Model systems that recapitulate the different aspects of neurodegenerative diseases are essential to understand the pathology, and to predict clinical efficacy during early development of new therapeutic approaches. Development of animal models for neurodegenerative diseases is a major challenge as the diseases have complex pathologies and symptoms can take decades to unfold. As a result, most of the currently available animal models for neurodegenerative diseases are not exact phenotypes of the human diseases and therefore lack the ability to forecast clinical success of new therapeutic approaches. Moreover, studies performed *in vitro* and *in vivo* on cells and small animals are difficult to translate to larger animals, because factors such as delivery route, dosing, distribution, and toxicity vary between the different model systems (Joshi et al., 2017).

### Animal Models for C9orf72 ALS/FTD

Various mouse models have been generated for *C9orf72* ALS/FTD that recapitulate distinct disease-related pathological, functional, and behavioral phenotypes. An overview of the currently available models is shown in Table 1. The first mouse model for *C9orf72* ALS was created in 2015 by somatic transduction of the C57BL/6J mouse CNS using an AAV carrying 66 G_4_C_2_ repeat (Chew et al., 2015). Although this model did not develop the severe ALS/FTD symptoms, some important features were observed that correlate with the pathology in patients. For example, sense RNA foci and DPRs produced from RAN translation were observed and TDP-43 inclusions were found in ∼7–8% of neurons in the cortex and hippocampus (Chew et al., 2015). In addition, mild neurodegeneration but no motor neuron loss was observed (Chew et al., 2015). Four other transgenic mouse models were created expressing either full length or truncated human *C9orf72* with the G_4_C_2_ expansion. The *C9orf72* Exon 1–6 BAC (G_4_C_2_)500 SJL/B6 mice expressed sense and antisense RNA foci and poly GP proteins but no motor and/or behavioral impairment was observed (Peters et al., 2015). The Tg(*C9orf72*_3) line 112 mouse was created containing the full length human *C9orf72* with multiple G_4_C_2_-repeat sizes ranging from 100 to 1000 repeats (O’Rourke et al., 2015). This model showed sense and antisense RNA foci and mild accumulation of poly GP but despite some evidence for nuclear stress there was no neuronal loss observed. Similarly, the BAC-C9-450 model also produced sense and antisense RNA foci in some brain regions and in the spinal cord as well as DPR proteins with an age dependent increase of poly(GA). Despite a partial learning deficit and increased anxiety, no other motor or behavioral changes were observed. In contrast, the FVB/NJ-Tg(*C9orf72*)500Lpwr/J mouse model expressing the full length mutated human *C9orf72* gene including the 52 kb 5′ upstream region and the 19 kb 3′ downstream region of the gene did show progressive neurodegeneration and decreased survival which is also seen in ALS patients (Liu et al., 2016). Sense RNA foci were observed in almost all NeuN positive neurons in the cortex, hippocampus and cerebellum. Antisense RNA foci were predominantly found in the motor cortex, hippocampus, cerebellar Purkinje layer and interneurons in the lateral and posterior horn of the spinal cord. DPR proteins in this mouse model increased with age throughout the brain, and nuclear and cytoplasmic TDP-43 aggregates were mainly observed in degenerating brain regions. The severe phenotype was exclusively observed in about one third of the females, while most of the males and females developed a mild phenotype. Nevertheless, the observations in this model strongly support the involvement of the G4C2 repeat-mediated gain of toxicity in the disease pathology.

**TABLE 1:** Overview of the currently available mouse models for *C9orf72* related ALS and/or FTD.

ALS mouse model	Mutation	Phenotype
AAV-G_4_C_2_66 (Chew et al., 2015)	(G_4_C_2_)_66_ + 119 bp 5′ + 100 bp 3′ region (expressed by CBA promoter)	Mild: histopathological features, Anxiety, decreased sociability, reduced motor function, weight loss, loss of NeuN positive neurons in cortex, motor cortex, Purkinje cells (Chew et al., 2015).
BAC-C9-500/300 (Peters et al., 2015)	Human *C9orf72* (exon 1–6) + ∼500 G_4_C_2_ repeats + 141 kb 5′ region	Only histopathological features (no TDP-43 pathology), no behavioral (Xu et al., 2013).
Tg(*C9ORF72*_3) Line 112 (BAC-C9-(100-1000)) (O’Rourke et al., 2015)	Human *C9orf72* (1–11) + mix of 100–1000 G_4_C_2_ repeats + 110 kb 5′ + 20 kb 3′ region	Only histopathological features (no TDP-43 pathology), no behavioral (O’Rourke et al., 2015).
BAC-C9-450 (Jiang et al., 2016)	Human *C9orf72* (exon 1–5) + ∼450 G_4_C_2_ repeats + 140 kb 5′ region	Mild: histopathological features, spatial learning deficit, anxiety, ∼10% loss of hippocampal neurons (Jiang et al., 2016).
BAC-C9-500 (Liu et al., 2016)	Human *C9orf72* (1–11) + ∼500 G_4_C_2_ repeats + 52 kb 5′ + 19 kb 3′ region	Severe: histopathological features, impaired motor function, reduced grip strength, hindlimb paralysis, decreased survival, loss of Purkinje cells, interneurons, upper and lower motor neurons (Liu et al., 2016).

Despite the variable penetrance, these models could still be valuable tools for research as they all display some specific features such as RNA foci and DPR proteins that are key characteristics of *C9orf72* related ALS/FTD patients.

### Animal Models for SCA3

More than 10 transgenic models for SCA3 have been published, all with variable differences in pathology and phenotype and most of them showing a mild form of neurodegeneration (Gould, 2012). A summary of the most commonly used mouse models and their key characteristics is shown in Table 2. Three models showed a severe phenotype and will be discussed here in more detail. The first transgenic mouse model for SCA3 was created more than two decades ago expressing either full length or truncated ataxin-3 with 79 CAG repeat (Q79). A severe ataxic phenotype was observed only in the truncated model, with degeneration of the cerebellum and loss of Purkinje cells (Ikeda et al., 1996). Due to this observation, it was strongly suggested that the C-terminal fragment of ataxin-3 containing the expanded polyQ could be more toxic by itself than when expressed as part of full length ataxin-3. Consistently, putative cleavage fragments of expanded ataxin-3 were identified in cell models, in post-mortem brain tissues of a SCA3 mouse model, in a drosophila model, and in patients (Yamamoto et al., 2001; Berke et al., 2004; Goti, 2004; Colomer Gould et al., 2006; Jung et al., 2009). It is now well-accepted that the gain of toxicity is directly caused by the CAG repeat and that the affected neurons express a protease that cleaves the mutant ataxin-3 protein, releasing short soluble polyQ fragments that are highly neurotoxic and more prone to form aggregates (Wang et al., 2011; Nalavade et al., 2013). Besides proteolytic cleavage of the C-terminal of the mutant ataxin-3 protein, mis-splicing could also play a role. A crucial evidence of mis-splicing leading to production of toxic short polyQ proteins was observed in HD. It was shown that exon 1 of the huntingtin, gene which contains the CAG repeat does not always splice to exon 2 but generates small polyadenylated HTTexon1 mRNA that encodes a small, highly pathogenic exon 1-polyQ HTT protein (also known as “exon-1 protein”) (Neueder et al., 2017).

**TABLE 2:** Overview of the currently available mouse models for SCA3.

SCA3 mouse model	Mutation	Phenotype
Q79 (Ikeda et al., 1996)	L7 promoter + Human Ataxin 3 + 79 CAG L7 promoter + truncated Human Ataxin 3 + 79 CAG	Severe when 79 CAG is expressed by itself: severe ataxia, gait disturbances, motor deficits (Ikeda et al., 1996).
MJD84.2 (Cemal, 2002)	Human ataxin-3 (YAC) + 84 CAG + 35 kb 5′ + 150 kb 3′	Intermediate: gait abnormalities, hypoactivity, Limb clasping, atrophy of the cerebellar Purkinje and molecular cell layers (Cemal, 2002).
Homozygous Q71C (Goti, 2004)	Mouse prion promoter + human ataxin-3 + 71 CAG	Severe: progressive postural instability, gait and limb ataxia, weight loss, premature death, neuronal intranuclear inclusions, decreased TH-positive neurons in the substantia nigra (Goti, 2004).
70.61 (Bichelmeier et al., 2007)	Mouse prion promoter + Ataxin-3 + 70 or 148 CAG	Severe: intranuclear inclusions in cortex and cerebellum, Shrinkage of ∼50–80% of Purkinje cells, premature death (Habig et al., 2007).
Ataxin-3-Q79HA (Chou et al., 2008)	Mouse prion promoter + Ataxin-3 + 79 CAG	Intermediate: neuronal dysfunction, ataxia, downregulation and upregulation of several genes (Chou et al., 2008).
PrP/MJD77-het/hom (Boy et al., 2009)	Ataxin-3 + 77 CAG	Mild: cerebellar dysfunction, reduced anxiety, hyperactivity, impaired rotarod performance, weight loss (Boy et al., 2009).
HDProm-MJD148 (Boy et al., 2010)	Ataxin-3 + 148 CAG (expressed by Huntingtin promoter)	Mild: late onset symptoms, declined motor coordination after 1 year (Boy et al., 2010).
Hemi-CMVMJD94 (Silva-Fernandes et al., 2010)	Ataxin-3 + 94 CAG (expressed by CMV promoter)	Mild: represents early disease symptoms, neuronal atrophy and astrogliosis in several brain regions (Silva-Fernandes et al., 2010).
Lentiviral Atx3-72Q (Nóbrega et al., 2013a)	Ataxin-3 + CAG (expressed by PGK promoter)	Mild: reduced motor coordination, wide-based ataxic gait, and hyperactivity. accumulation of intranuclear inclusions, neurodegeneration (Nóbrega et al., 2013a).
Humanized SCA3 knockin (Ki91) (Switonski et al., 2015)	Ataxin-3 + 91 CAG	Mild: deficits in coordination, transcriptional changes in the brain, amyloid depositions, mild degeneration of Purkinje cells in older mice, increased GFAP-positive glia in cerebellar white matter (Switonski et al., 2015).

The two other SCA3 mouse models (Q71C and 70.61) with a severe phenotype both express mutant ataxin-3 under control of the prion protein promoter. Within 1 to 8 months, both models showed early onset and a rapidly progressive type of SCA3 with several abnormalities such as progressive neurodegeneration, weight loss, behavioral problems, neuronal inclusions and premature death. Taken together, all these models could be useful to further study the disease mechanism in SCA3 and serve as tools for preclinical studies targeting mutant ataxin-3. It should be noted that the models with a severe phenotype all use non-native promoters to drive expression of mutant ataxin-3, lack regulatory flanking sequences, and often contain an excessive number of transgene copies. Thus, these models do not exactly mimic the expression pattern of mutant ataxin-3 in patients.

## RNAi-Based Therapeutic Strategies for Neurodegenerative Diseases

As discussed, several neurodegenerative diseases are caused by mutations that lead to gain of toxic functions, and gene silencing technologies are attractive to lower the expression of disease-causing genes. A specific technology that can be applied for gene silencing isRNAi.

The discovery of RNAi dates back almost three decades, when Napoli (1990) reported that injection of an extra chimeric copy of chalcone synthase, a gene responsible for the purple pigment anthocyanin in petunias, unexpectedly resulted in white petunias. Thus, instead of complementing each other and producing extra purple flowers, the two copies of chalcone synthase seemed to interact with each other and turn themselves off. The mechanism underlying this observation remained unclear until, when Fire et al. (1998) provided an explanation of the RNAi mechanism. They discovered that a small non-coding RNA, miRNA (lin-4), binds to the 3′-UTR of the lin-14 mRNA in *C. elegans*, silencing its expression. For this work they were awarded the Nobel prize in Physiology or Medicine in 2006 (Fire et al., 1998). It was subsequently demonstrated that miRNA-mediated silencing also occurs in mammalian cells and this mechanism became a popular new tool to study gene function (Elbashir et al., 2001).

It is now known that RNAi is a naturally occurring process in eukaryotic cells where double stranded RNA molecules can knock down or suppress the expression of genes that contain a homologous RNAi target sequence. RNAi plays an important physiological role in gene regulation and also has a function in the innate immune response of cells by providing protection against foreign nucleic acids from pathogens such as viruses and bacteria (Ozcan et al., 2016). One of the first discovered mediators of RNAi is the RNAi-induced silencing complex (RISC), which has a nuclease activity that can cleave mRNA and knock down its expression. RNAi can be triggered by both endogenous and exogeneous double stranded RNAs (Baulcombe, 1996; Montgomery and Fire, 1998; Saito et al., 2015). Three types of small non-coding RNAs use the RNAi pathway; microRNA (miRNAs), small interfering RNA (siRNA), and piwi-interacting RNAs (piRNAs). miRNAs and siRNAs have a role as negative regulators of gene expression, while the piRNAs defend organisms against transposable elements (Aravin et al., 2007). miRNAs and siRNA are currently both being widely used as therapeutics in clinical trials and each system has its own merits.

### miRNAs and Their Processing

miRNAs are small non-coding RNAs that are thought to regulate about 30% of genes in humans. More than 2000 miRNAs^1^ have been identified in humans and these are known to regulate several important cellular processes. Dysregulation of miRNAs is associated with several types of metabolic and CNS diseases, and with cancer. Apart from regulating gene expression, miRNAs can act as signaling molecules for intercellular communication. For example, it has been shown that miRNAs can be packaged into exosomes or microvesicles that, following secretion, can be endocytosed by secondary cells, and re-establish their function (Kreth et al., 2018).

**FIGURE 4:**
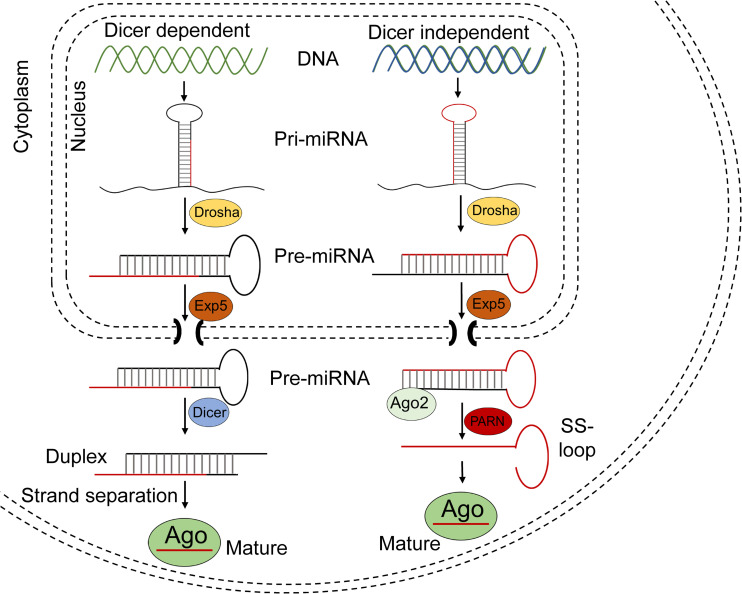
Schematic of miRNA processing pathway. Most miRNAs are processed in the Dicer dependent (canonical) pathway shown in the left part of the figure. After being transcribed, the pri-miRNA transcripts are cropped by Drosha in the nucleus, resulting in a 60–70 nt pre-miRNA. The pre-miRNA is exported to the cytoplasm by Exportin 5 and the hairpin is then diced by Dicer into ∼22-nt miRNA duplex, after which it is separated into a guide and passenger strand. The guide strand is usually loaded into Ago proteins to form RISC.

**FIGURE 5:**
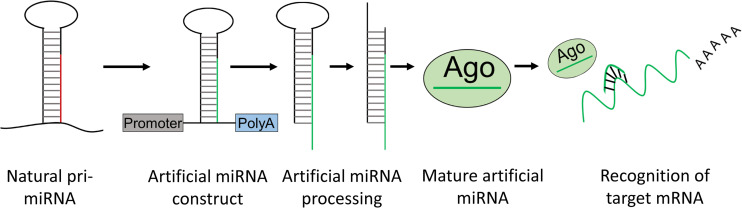
Artificial miRNA design. Artificial miRNAs can be made by replacing the mature miRNA sequence of a natural pri-miRNA for a complementary sequence of a target mRNA of interest. The artificial pri-miRNA sequence can be cloned in an expression construct. Upon transfection with the artificial miRNA construct, the artificial miRNA is processed in the cell into a mature artificial miRNA which can bind and knockdown expression of an mRNA of interest.

The biogenesis of a miRNA is tightly regulated in humans and involves 4 key enzymes: Drosha, exportin 5, Dicer, and argonaute (AGO) proteins (Figure 4). The precursor of a miRNA is naturally encoded in the genome and is transcribed by RNA polymerase II or III into a long primary miRNA (pri-miRNA) with a cap and poly-A tail. The pri-miRNA transcript folds into a complex hairpin structure consisting of a double-stranded stem of about 30 bp, a terminal loop and two flanking unstructured single-stranded tails. The pri-miRNA is further processed in the cell nucleus by a ribonuclease called Drosha, resulting in a short 70-nt stem–loop structured precursor miRNA (pre-miRNA). The pre-miRNA is then transported to the cytoplasm by exportin 5 and is cleaved into a short double stranded miRNA by an RNAse III enzyme called Dicer. The double stranded miRNA is recognized by AGO proteins and is loaded on RISC, usually preserving the guide strand and degrading the passenger strand. The RISC-AGO complex guides the miRNA guide strand to its target mRNA and induces its degradation and/or inhibits its translation. The first seven nucleotides near the 5′ end of the guide strand form the seed sequence and are critical for the target recognition. It is also worth mentioning that not all miRNAs are processed as described above and miRNA processing in plants differs at several points from the above described pathway. Furthermore, several mammalian miRNAs can bypass Drosha and currently one miRNA is known to bypass Dicer processing (Yang and Lai, 2011). This Dicer-independent miRNA is microRNA-451a (miR-451a or miR-451) and has a role in the regulation of erythroid development (Herrera-Carrillo and Berkhout, 2017). Its recognition by Dicer seems to be perturbed due to its unusually short base paired stem and as a result, an active strand derived from the stem is produced, consisting of the single-stranded loop and part of the complementary stem region (Figure 4). The ability to design artificial miRNAs that specifically silence disease-related genes is the basis for therapeutics such as miRNA mimics, anti-miRs, and artificial miRNAs. miRNA mimics are double stranded miRNAs made synthetically to match a corresponding miRNA aiming to compensate the loss of miRNAs that are downregulated in diseases. Anti-miRs are artificially made single stranded miRNA used to bind target miRNAs and inhibit their function. Artificial miRNAs are made by replacing the guide strand sequence of an endogenous miRNA precursor with the sequence of an mRNA of interest, enabling silencing of genes that are upregulated in diseases (Figure 5). More recently, miR-451 was discovered that follows a non-canonical processing of miR-451 to silence genes involved in diseases (Cheloufi et al., 2010; Cifuentes et al., 2010; Yang et al., 2010; Herrera-Carrillo and Berkhout, 2017). miR-451 produces no passenger strand as the pre-miR-451 escapes Dicer cleavage and only a 5′ arm strand is active on the targets. Thus, there is no miRNA duplex formation that requires strand separation and selection for the RISC. Because of this efficient processing, off-target effects due to a passenger strand activity can be neglected.

There are four Ago proteins and all are capable to repress translation or promote mRNA degradation. Ago2 is the only Ago protein with a slicer activity which plays a critical role in Dicer independent processing of miR451 (right part of the figure). miR451 is also processed in the nucleus by Drosha, but results in an unusually short 41–42-nt pre-miRNA which is not recognized by Dicer and does not form a miRNA duplex. However, the further processing of miR-451 requires the slicer activity of Ago2. Ago2 cleaves the 3′ arm of pre-miRNA by its slicer activity and yields a 30-nt intermediate whose 3′ end is further trimmed by PARN to generate a mature ∼23-nt miRNA. This mature miRNA is loaded into Ago proteins to form the RISC.

### Clinical Applications for Therapeutic RNAi

Several RNAi-targeted therapeutics have reached clinical development. An example of this is MRX34 (synthetic miR-34), a miRNA developed by Mirna Therapeutics which was the first to enter a clinical trial to treat different types of cancer (Beg et al., 2016, 2017). Its mode of action was to decrease the expression of collagen and other proteins that are involved in fibrous scar formation. However, clinical development was terminated in 2016 due to several immune-related severe adverse events. An example of a successful anti-miR clinical trial is Miravirsen, a Locked Nucleic Acid (LNA) anti-miR-122 oligo developed by Santaris Pharma to treat Hepatitis C (Gebert et al., 2014). The phase II trials demonstrated reduced Hepatitis C viral load, even at low therapeutic concentrations and no adverse events were observed (Chang et al., 2008; Henke et al., 2008; Tiemann and Rossi, 2009).

Furthermore, Alnylam Pharmaceuticals was recently granted marketing authorization after a successful phase III clinical trial for GIVLAARI^®^ (givorisan), an aminolevulinate synthase 1 (ALAS1)-directed small interfering siRNA for treatment of Acute Hepatic Porphyria. Givorisan is injected subcutaneously once a month and its mode of action is to reduce elevated levels of ALAS1 mRNA and prevent accumulation of neurotoxic δ-aminolevulinic acid and porphobilinogen levels that are associated with acute porphyria attacks. The clinical trial demonstrated a 74% reduction of porphyria attack in patients, despite some frequently occurring adverse reactions such as injection site reaction, nausea, and fatigue (Alnylam Pharmaceuticals, 2020).

Regulus therapeutics also have several RNAi-based therapeutics in the pipeline and currently RG-012 is in Phase II clinical trial. RG-012 is a single stranded, chemically modified oligonucleotide that binds to and inhibits the function of miR-21 for the treatment of Alport syndrome. Patients with Alport syndrome experience progressive loss of kidney function due to defective mutations in collagen genes which are normally responsible for maintaining the integrity and structure of the kidneys. MiR-21 represses expression of proteins in the PPARα signaling pathway which have a protective function in the kidneys. In preclinical studies, RG-012 showed strong inhibition of miR-21 and resulted in improvement of kidney function and an increased life expectancy (Chau et al., 2012).

Another phase I/II clinical trial to treat patients with Hepatitis C infection based on shRNAs was conducted by Benitec Biopharma. Their lead product was TT-034 which is based on intravenous AAV8 delivery of three independent shRNAs targeting three well-conserved sequences located in the 5′ UTR and NS5B regions of the HCV genome (Patel et al., 2016). Overall, TT-034 was well-tolerated and safe in patients. Furthermore, liver biopsies revealed sustained transduction of hepatocytes and expression of the three anti-HCV shRNAs. However, Benitec Biopharma decided to discontinue this program due to decrease in commercial opportunities for TT-034 following the introduction of highly effective viral eradication strategies based on (combinations of) small molecules.

Compared to miRNAs, siRNAs have been more widely tested in clinical trials for treatment of different types of diseases such as cancer, viral infections, age-related macular degeneration, diabetic macular, hypercholesterolemia, and ocular hypertension (Chakraborty et al., 2017). DNA constructs encoding therapeutic RNAi following delivery by lentiviral vectors have also been clinically tested. Benitec, Inc. in collaboration with the City of Hope National Medical Center conducted a clinical trial to treat HIV-1 infection in AIDS/lymphoma patients (DiGiusto et al., 2010). This was executed by genetically modifying hematopoietic stem cells (hSC) *ex vivo* by transduction with a lentiviral vector expressing three small RNAs targeting HIV: an shRNA targeting an exon in HIV-1 tat/rev, a RNA decoy for the HIV TAT-reactive element, and a ribozyme targeting the host cell CCR5 chemokine receptor. The transduced hSC were then infused into patients whose bone marrow had been ablated to treat their AIDS related lymphoma. Sustained expression of the anti-tat/rev shRNA and ribozyme was observed for up to 24 months post-infusion (Tiemann and Rossi, 2009). Although this study demonstrated feasibility and safety of this approach, it failed to demonstrate clinical benefit because an insufficient number of hSCs were transduced.

In the neurodegenerative field, uniQure obtained Food and Drug Administration (FDA) clearance for AMT-130 which is the first AAV-miRNA-based gene therapy for a neurodegenerative disease to start a Phase I/II clinical trial. Recently, uniQure announced that the first two patients have been treated in this double-blind, randomized clinical trial being conducted in the United States. AMT-130 is based on a miRNA designed to target both wild-type and mutant Huntingtin allele (AAV5-miHTT) (Miniarikova et al., 2016, 2017, 2018; Evers et al., 2018; Keskin et al., 2019). Significant lowering of human mutant huntingtin mRNA and protein was achieved in the brain of a transgenic HD (tgHD) minipig model after a single administration into the brain (Evers et al., 2018). The Phase I/II clinical trial of AMT-130 for the treatment of HD will explore the safety, tolerability, and efficacy signals in 26 patients with early manifest Huntington’s disease randomized to treatment with AMT-130 or an imitation (sham) surgery. The 5-year, multi-center trial consists of a blinded 18-month core study period followed by unblinded long-term follow-up. Patients will receive a single administration of AMT-130 through MRI-guided, convection-enhanced stereotactic neurosurgical delivery directly into the striatum (caudate and putamen). Additional details are available on www.clinicaltrails.gov (NCT04120493). A similar approach is being pursued by Voyager Therapeutics and promising results have been obtained with their lead candidate VY-HTT01 in preclinical models. miRNA mediated silencing is thought to be an attractive therapeutic modality in many other neurodegenerative diseases, including the SCAs, ALS, FTD, synucleopathies including Parkinson’s disease, tauopathies and AD (Xu et al., 2005; Do Carmo Costa et al., 2013; Liu et al., 2013; Nóbrega et al., 2013b). miRNAs may cause toxicity related to (passenger strand) off-target toxicity. Hence, during development these effects should be closely investigated and minimized, by selecting scaffolds with little to no passenger strand activity and by predicting the chances of binding to off-target genes using computer-based bioinformatic tools. Another important limitation of miRNA-based therapeutics is the required efficacy in the nucleus of cells due to the fact that active mature miRNAs resulting from canonical Dicer processing in the cytoplasm are thought not to re-enter the nucleus. However, as many of these hurdles are being tackled, more miRNA therapeutics are expected to reach the clinic.

## Common Alternative Silencing Strategies for Neurodegenerative Diseases

### Antisense Oligonucleotides

Antisense oligonucleotides (ASOs) are short, synthetic, single-stranded nucleic acids of about 20–25 bases long that bind cellular RNA and reduce, restore, or modify protein expression via several distinct mechanisms. The silencing pathway of ASOs differs from siRNA and miRNAs and there is no known cellular mechanism to facilitate strand recognition. ASOs classically bind to complementary mRNA through Watson Crick base-pairing leading to endonuclease-mediated transcriptional knockdown (Rinaldi and Wood, 2018). Once bound to the mRNA ASOs can form an RNA–DNA hybrid that becomes a substrate for the enzyme RNase H, which hydrolyzes the mRNA resulting in its degradation (Wu et al., 2004). The cleaved mRNA products are then processed by the normal cellular degradation pathways in the nucleus and cytoplasm (Lima et al., 2016). ASOs can be engineered to have enhanced pharmacological properties by introducing backbone modifications. For example, modified ASOs can knockdown gene expression by sterically blocking splicing factors and altering pre-mRNA splicing, or by preventing ribosome recruitment to block mRNA translation (Havens and Hastings, 2016). Interestingly, ASOs can destabilize splicing sites and displace or recruit splicing factors when designed to bind within intron–exon junctions. This makes it possible to exclude (“skip”) or include exons of interest, which can be beneficial in genetic diseases (Havens and Hastings, 2016). Through this mechanism normal gene function can be restored by re-establishing a normal reading frame following a pathogenic frame shift, or by excluding mutated segments of DNA (Rinaldi and Wood, 2018). ASOs have been used in preclinical studies since the 1970s and several programs have made it into clinical development. A phase I ALS trial using intrathecal administration of ASOs targeting SOD1 was completed in 2012 by Ionis Pharmaceuticals (Table 3) (Miller T. et al., 2013; Miller T.M. et al., 2013). Unfortunately, this study failed to show reduction of SOD1 protein which was explained by the low target tissue ASO concentration. However, the treatment proved to be safe, and a follow up phase I/II trial study is ongoing (BIIB067; IONIS-SOD1). Ionis Pharmaceuticals also initiated a phase I clinical trial in 2019 with ASOs targeting *C9orf72*-ALS patients (BIIB078). BIIB078 is targeting specifically the intronic region of *C9orf72* to cause reduction of only the repeat-containing *C9orf72* transcripts and is administered intrathecally to adult patients. Ionis Pharmaceuticals has developed ASOs for many other diseases and have ongoing clinical trials for HD, myotonic dystrophy type 1 (DM1) and spinal muscular atrophy (SMA). Furthermore, several preclinical studies have shown efficacy of ASOs in *C9orf72*-related ALS and SCA3 *in vitro* and *in vivo* but these programs have not yet reached clinical development. A clinical study (NCT03508947) with ASOs that has raised some concerns regarding toxicity was performed by WAVE life Sciences to treat patients with Duchenne muscular dystrophy (DMD). DMD is a fatal genetic disorder characterized by progressive muscle wasting and is caused by mutations in the DMD gene which encodes for dystrophin, an essential protein to maintain muscle integrity. The ASO was administered intravenously and was designed to skip the mutated exon 51 in the DMD gene to restore normal function of dystophin (Echevarría et al., 2018). Adverse events such as headache, fever, vomiting, and tachycardia occurred in the low and mid dose groups and these adverse events were even more severe in the high-dosed groups. WAVE life sciences reported plans to continue with the lower doses for Phase II/III trial but it is unclear if these adverse events will re-occur during re-administration and whether or not the lower doses are therapeutically effective. A major limitation of ASOs for clinical application is that they are degraded by endo- and exo-nucleases and re-administration is required (Evers et al., 2015). This is especially problematic in CNS disorders that require invasive delivery methods. However, the pharmacological profiles of ASOs can be enhanced by introducing chemical modifications and efforts are currently being made to improve ASOs delivery to for example cross the blood–brain barrier (BBB) and to improve target engagement (Juliano, 2016).

**TABLE 3:** Clinical trials using ASOs for ALS by Ionis Pharmaceuticals.

Disease	Trial code	Vector	Delivery route	Status (completion year)	Sponsor
ALS (Miller T. et al., 2013; Miller T.M. et al., 2013)	NCT01041222	ASO (SOD1)	Intrathecal	Phase I (2012)	Ionis Pharmaceuticals
ALS	NCT02623699	ASO (SOD1)	Intrathecal	Phase I/II (2019)	Biogen & Ionis Pharmaceuticals
ALS	NCT03626012	ASO (*C9orf72*)	Intrathecal	Phase I (2022)	Biogen & Ionis Pharmaceuticals

### Gene Editing (CRISPR)

Gene editing is a relatively novel approach to remove, add, or alter DNA in a sequence-specific manner. The pivotal discovery that made gene editing possible was the discovery that the endogenous cellular repair machinery can be triggered by targeted DNA double strand breaks (DSBs) through homology-directed repair (HDR) or non-homologous end-joining (NHEJ). DNA DSBs can be introduced at a precise location by using engineered nucleases harboring a sequence-specific DNA-binding domain, fused to a DNA cleavage module. The most popular nucleases are zinc-finger nucleases (ZFNs), transcription activator-like effector nucleases (TALENs), and the RNA-guide clustered regulatory interspaced short palindromic repeats (CRISPR) and CRISPR-associated system 9 (Cas9) (CRISPR/Cas9). ZFNs are currently the only gene editing technology that have made it to clinical trials. In 2018, Sangamo Therapeutics started a clinical trial using this technology in mucopolysaccharidosis II (MPS II). ZFNs consist of ∼30 amino acids which folds into ββα “fingers” structures that recognize and bind a trinucleotide sequence of DNA (Gupta and Musunuru, 2014; Corrigan-Curay et al., 2015; Nature America, Inc, 2018). The nuclease domain is based on a restriction endonuclease (*Fok*I) that can cut DNA following dimerization (Gupta and Musunuru, 2014). Binding to DNA sequences longer than 3 and up to 18 bp (in multiples of 3 bp) is possible by arranging a series of linked zinc-fingers. However, this requires recoding of proteins for each new target site which is a very challenging and time-consuming process. Another limitation is the restricted target site selection, as zinc-fingers can only target binding sites every ∼50–200 bp in a random DNA sequence (Gupta and Musunuru, 2014). Furthermore, all gene editing technologies including zinc-fingers may have off-target effects, resulting in unwanted induction of DNA mutations or deletions. Like ZFNs, TALENs are based on a DNA–protein association but they use a different DNA binding domain termed (transcription activator-like effectors) TALE repeats (Gupta and Musunuru, 2014). A TALE motif can recognize a single nucleotide and an array of TALEs can recognize and bind to a longer DNA sequence. Unlike ZFNs, the design of TALENs are less complicated and the TALE repeat array can be easily extended to whatever length is desired (Gupta and Musunuru, 2014). Furthermore, as compared to ZFNs, site-specific targeting is easier because multiple TALEN pairs are available for each base pair of a random DNA sequence. Some major limitations for TALENs are off-target effects, and compared to ZFNs they are about 3x larger in size (∼3 kb) which makes delivery by viral vectors more challenging.

CRISPR/Cas9 is the latest of the above-mentioned nuclease systems and is based on a naturally occurring process in the adaptive immune system of the bacteria *Streptococcus* pyogenes. CRISPR/Cas9 is distinct from ZFN and TALEN endonucleases in that it does not use a protein but an RNA-guided system to perform genome editing. Unlike the other nuclease systems, CRISPR/Cas9 does not require custom design of novel proteins for each DNA target site. As a result, the design of constructs is relatively easy and cost-effective. Bacteria use the RNA-guided Cas proteins to create DSBs in exogenous DNA derived from invading viruses (bacteriophages). It does so by creating CRISPR arrays from exogenous DNA by insertion of these DNA sequences between short palindromic repeats in the CRISPR locus of its own DNA. Upon a repeated viral attack, these “foreign” inserts can be transcribed from the virus specific CRISPR locus into CRISPR RNA fragments that match the invading viral DNA. CRISPR RNAs contain a tail transcribed from the CRISPR locus which facilitates incorporation into complexes to form hairpin-like structures that allow them to dock on a Cas protein. The Cas protein is then guided by the CRISPR RNA to hybridize to its parental exogeneous DNA and induce a DSB (Cong and Zhang, 2014). This CRISPR/Cas9 system can be implemented in eukaryotes by designing sequences that target a specific DNA sequence and co-expressing Cas9. The Cas9 protein cuts the DNA at the target site, which is repaired by HDR or NHEJ. Several animal models have been created using the CRISPR/Cas9 system and a rapidly increasing number of preclinical studies shows the promise of this technology in combination with gene therapy for the treatment of neurodegenerative diseases. For example, in AD, the defective (amyloid precursor protein) APP gene was successfully edited in human fibroblasts using a CRISPR/cas9 construct and this resulted in a reduction of the levels of Amyloid beta, a main component of plaques found in the brains of AD patients (Gyorgy et al., 2016). In ALS, the G_4_C_2_ repeat in the non-coding region of the *C9orf72* gene was successfully deleted in transfected patient-derived iPS cells and this prevented RNA foci formation as well as the promoter hypermethylation that is typical for ALS (Pribadi et al., 2016). For HD, permanent suppression of mutant Huntingtin and it’s aggregates was achieved in the striatum of the HD140Q-knock-in mice by CRISPR/Cas9 (Yang et al., 2017). For SCA3, CRISPR/Cas9-mediated deletion CAG repeat in ATXN3 gene was successfully performed in patient-derived iPSCs (Ouyang et al., 2018). Following the CAG deletion, the iPSCs retained pluripotency and neurons differentiated from these cells retained a normal ubiquitin-binding capacity of ATXN3.

The CRISPR/Cas9 system has great potential for targeting pathogenic genes, but major hurdles are the occurrence of off-target effects, Cas9 specificity, and potential mutagenesis. Not unexpectedly, continuous expression of Cas9 proteins at high concentrations has been linked to toxicity (Morgens et al., 2017). The chances for off-target binding in the host genome using current systems are estimated to be relatively high (≥50%) (Zhang X.H. et al., 2015). Another limitation is the delivery method as the CRISPR/Cas9 components are about 8–10 kb long whereas AAV vectors have a packaging capacity limited to 4.8 kb. Thus, the CRISPR/Cas9 system has a huge potential to be developed in therapeutic approaches but major challenges need to be overcome to make this technology suitable to treat human diseases.

## AAVs for Gene Transfer to the Nervous System

A major difficulty to treat neurodegenerative diseases remains the method of administration. Due to existence of the BBB, many small molecules and most therapeutic nucleotides or gene therapy vectors that are administered systemically or orally fail to reach the brain or spinal cord at a therapeutically relevant dose. Different alternative routes of administration are being investigated for delivery to the CNS and their application is highly dependent on the disease pathology. While some diseases require local targeting or transduction, others require widespread distribution throughout the CNS. Intracerebral administration, directly injected in the parenchyma of the brain can be safely and effectively done using convection-enhanced delivery (CED) in combination with precise positioning using magnetic resonance imaging-based guidance technologies (MRI) (Salegio et al., 2012). CED uses a pressure gradient to generate bulk flow within the brain parenchyma. Due to the low velocity of injection potential structural damage is minimized and a uniform distribution can be obtained (Bankiewicz et al., 2000; Richardson et al., 2011). The advantage of this injection route is that it bypasses the BBB and high local transduction can be obtained with a relatively low dose of AAV and limited leakage to periphery organs. Further spread of AAV may occur via anterograde or retrograde transport along axons but this is highly dependent on the AAV serotype (Aschauer et al., 2013; Warren Olanow et al., 2015). A drawback of this delivery route is that the procedure itself is invasive and may lead to complications such as bleeding or leakage of the administered vector into the CSF. In addition, intracerebral administration is only suitable for diseases with pathology limited to specific brain areas such as HD. In the current AMT-130 clinical trial by uniQure, the vector is delivered directly into the striatum (caudate and putamen) using MRI-guided, convection-enhanced stereotactic neurosurgery. This approach has been shown to be very precise and safe for direct infusion into the brain (Samaranch et al., 2017). Alternative routes used to cover larger areas of the CNS are systemic or intrathecal delivery. Systemic delivery by intravenous injection is a relatively simple procedure, less invasive and avoids costly neurosurgical procedures. However, systemic delivery of AAV gene therapy is currently less suitable for neurodegenerative disorders because the bulk of such vectors is taken up by peripheral organs and can cause systemic immunogenicity. Even though some vectors can cross the BBB, the target tissue concentration after systemic delivery is often not therapeutically relevant. Nevertheless, a successful clinical trial with systemic administration was performed by AveXis to treat children with SMA (Mendell et al., 2017). The vector used was a self-complementary AAV9 and following systemic administration a therapeutic effect in motor neurons in the spinal cord was observed. It remains questionable whether similar effects can be obtained in adults as the integrity of the BBB is known to be different. In addition to systemic administration, AveXis is currently performing another clinical trial in children with SMA by injecting intrathecally. In non-human primates (NHP), they showed improved transduction of the CNS with up to 10 times lower intrathecal dose as compared to intravenous administration (Meyer et al., 2015). Intrathecal administration can be done through lumbar puncture, direct administration into the cisterna magna or in the cerebral ventricle (Hocquemiller et al., 2016; Hinderer et al., 2017). Thus, the vector is delivered directly into the CNS and bypasses the BBB. This approach is less invasive than intracerebral administration and usually shows less leakage to the periphery organs compared to systemic administration. One limitation of intrathecal administration is dilution of vector and the consequent transduction is usually lower when compared to intracerebral administration. Furthermore, transduction of the deeper brain structures is poor, possibly because the vector needs to pass the ependymal cell layer or the pia mater (Dindot et al., 2011; Mittermeyer et al., 2012).

## Gene Therapy Clinical Trials for Neurodegenerative Diseases or Neuromuscular Diseases

Several gene therapies for different types of neurodegenerative diseases have progressed into clinical development. For *C9orf72* related ALS and SCA3, no gene therapy has been clinically investigated yet. An overview of the currently ongoing clinical trials published on https://clinicaltrials.gov is depicted in Table 4 and will be further discussed in this paragraph.

**TABLE 4:** Gene therapy clinical trials for neurodegenerative/neuromuscular diseases.

Disease/condition	Trial code	Vector	Delivery route	Status (completion year)	Sponsor
AADC deficiency	NCT01395641	AAV2-hAADC	Intraparenchymal (Putamen)	Phase I/II (2020)	National Taiwan University Hospital
AADC deficiency	NCT02926066	AAV2-hAADC	Intraparenchymal (Putamen)	Phase II (2018)	National Taiwan University Hospital
AADC deficiency	NCT02852213	AAV2-hAADC	Substantia nigra pars compacta + ventral tegmental area	Phase I (2021)	Krystof Bankiewicz, University of California, San Francisco
Aging	NCT04133649	AAV-hTERT	Intravenous	Phase I (2021)	Libella Gene Therapeutics
Alzeimer	NCT00087789	AAV2-NGF	Basal forebrain	Phase 1 (2010)	Ceregene
Alzeimer (Rafii et al., 2018)	NCT00876863	AAV2-NGF	Basal forebrain	Phase II (2020)	Sangamo Therapeutics
Alzeimer	NCT03634007	AAVrh.10hAPOE2	Intracisternal	Phase I (2021)	Weill Medical College of Cornell University
Alzeimer	NCT04133454	AAV-hTERT	intravenously + intrathecal	Phase I (2021)	Libella Gene Therapeutics
Batten (Schulz et al., 2013; Drack et al., 2015; Foust et al., 2016)	NCT03770572	AAV9-CLN3	Intrathecal	Phase II (2023)	Amicus Therapeutics
Batten (Dyke et al., 2008)	NCT00151216	AAV2CUhCLN2	Intracranial	Phase I (2020)	Weill Medical College of Cornell University
Batten	NCT01161576	AVRh.10CUhCLN2	Intracranial	Phase I (2020)	Weill Medical College of Cornell University
Batten	NCT02725580	scAVV9.CB.CLN6	Intrathecal	Phase II (2019)	Nationwide Children’s Hospital
Batten disease	NCT01414985	AAVrh.10CUCLN2	Intracranial	Phase I/II (2024)	Weill Medical College of Cornell University
Canavan disease (Leone et al., 2012)	NA	AAV2-hASPA	Intraparenchymal	Phase I	National Institute of Neurological Disorders and Stroke
Duchenne muscular dystrophy (Mendell et al., 2010; Bowles et al., 2012)	NCT00428935	rAAV2.5-CMV-dystrophin	Intramuscular	Phase I (2010)	Nationwide Children’s Hospital
Duchenne muscular dystrophy	NCT02354781	rAAV1.CMV.huFollistin344	Intramuscular	Phase I/II (2017)	Jerry R. Mendell, Nationwide Children’s Hospital
Duchenne muscular dystrophy	NCT03368742	AAV9-dystrophin	Intravenous	Phase I/II (2024)	Solid Biosciences, LLC
Duchenne muscular dystrophy	NCT03362502	AAV9-dystrophin	Intravenous	Phase I (2025)	Pfizer
Gaucher disease (type 2)	NCT04411654	AAV9-GBA1	Intracisternal	Phase I/II (2027)	Prevail Therapeutics
Huntington’s disease	NCT04120493	AAV5-miHTT	Striatum	Phase I/II (2026)	uniQure Biopharma B.V.
MPS I (Harmatz et al., 2018)	NCT02702115	AAV6-ZFN	Intravenous	Phase I/II (2022)	Sangamo Therapeutics
MPS II (Laoharawee et al., 2018)	NTC03041324	AAV6-ZFN	Intravenous	Phase I (N.A)	Sangamo Therapeutics
MPS IIIA	NCT02716246	AAV9-hSGSH	Intravenous	Phase I/II (2019)	Abeona Therapeutics
MPS IIIA (Vincent et al., 2014)	NCT03612869	AAVrh.10-hSGSH	Intracerebral	Phase II/III (2022)	LYSOGENE
MPS IIIA	NCT04088734	scAAV9.U1a.hSGSH	intravenous	Phase I/II (2022)	Abeona Therapeutics, Inc
MPS IIIA (Vincent et al., 2014)	NCT03612869	AAVrh10-h.SGSH	Intracerebral	Phase III (2022)	LYSOGENE
MPS IIIB	NCT03315182	AAV9-hNAGLU	Intravenous	Phase I/II (2020)	Nationwide Children’s Hospital
Parkinson (Marks et al., 2008)	NCT00252850	AAV2-Neurturin	Intrastriatal	Phase I (2007)	Ceregene
Parkinson (Warren Olanow et al., 2015)	NCT00400634	AAV2-Neurturin	Putamen	Phase II (2008)	Ceregene
Parkinson (Bartus et al., 2013)	NCT00985517	AAV2-Neurturin	Substantia nigra	Phase II (2018)	Sangamo Therapeutics
Parkinson (Björklund et al., 2000; Airaksinen and Saarma, 2002; Bäckman et al., 2006)	NCT01621581	AAV2-GDNF	Putamen	Phase I (2022)	National Institute of Neurological Disorders and Stroke (NINDS)
Parkinson (Christine et al., 2009)	NCT00229736	AAV-hAADC-2	Striatum	Phase I (2013)	Genzyme/Sanofi
Parkinson	NCT01973543	AAV2-hAADC	Putamen	Phase I (2020)	Neurocrine Biosciences
Parkinson	NCT03562494	AAV2-hAADC	Putamen	Phase II (2020)	Neurocrine Biosciences
Parkinson	NCT02418598	AAV-hAADC-2	Putamen	Phase II (2018)	Jichi Medical University
Parkinson (Palfi et al., 2014)	NCT01856439	ProSavin (LV-TH-GCH-AADC)	Intrastriatal	Phase I/II (2021)	Oxford BioMedica
Parkinson	NCT03720418	AXO-Lenti-PD(OXB-102-01)	Intrastriatal	Phase I/II (2020)	Axovant Sciences Ltd
Parkinson (Kaplitt et al., 2007; LeWitt et al., 2011)	NCT00643890	AAV-GAD	Subthalamic nucleus	Phase II (terminated)	Neurologix, Inc
Parkinson (Marks et al., 2008)	NCT00252850	AAV2-NTN	Striatum	Phase I (2007)	Ceregene
Idiopathic Parkinson (Marks et al., 2010)	NCT00400634	AAV2-NTN	Putamen	Phase II (2008)	Ceregene
Parkinson	NCT00985517	AAV2-NTN	Substantia + putamen	Phase II (2017)	Sangamo Therapeutics
Pompe disease	NCT04174105	AAV8-GAA	Intravenous	Phase II (2026)	Audentes Therapeutics
Pompe disease	NCT04093349	AAV-GAA	Intravenous	Phase II (2023)	Spark Therapeutics
Pompe disease (Han et al., 2017)	NCT03533673	AAV2/8LSPhGAA	Intravenous	Phase II (2020)	Asklepios Biopharmaceutical, Inc.
Pompe disease	NCT02240407	rAAV9-DES-hGAA	Intramuscular	Phase I (2021)	University of Florida
Pompe disease (Cleaver et al., 2013)	NCT00976352	rAAV1-CMV-GAA	Intradiaphragmatic	Phase II (2015)	University of Florida
Sanfilippo disease type A	NCT02053064	AAVrh10-SGSH	Intracerebral	Phase II (2017)	LYSOGENE
Sanfilippo type B (Ellinwood et al., 2011)	NCT03300453	rAAV2/5-hNAGLU	Intracerebral	Phase I/II (2019)	uniQure Biopharma B.V.
Spinal muscular atrophy type 1 (Bevan et al., 2010, 2011)	NCT02122952	AAV9-SMN	Intravenous	Phase I (2017)	AveXis, Inc.
Spinal muscular atrophy (Duque et al., 2015; Meyer et al., 2015)	NCT03505099	AAV9-SMN	Intravenous	Phase III (2020)	AveXis, Inc.
Spinal muscular atrophy (Duque et al., 2015; Meyer et al., 2015)	NCT03306277	AAV9-SMN	Intravenous	Phase I (2019)	AveXis, Inc.
Spinal muscular atrophy type 1 (Mendell et al., 2017)	NCT03461289	AAV9-SMN	Intravenous	Phase III (2020)	AveXis, Inc.
Spinal muscular atrophy (Mendell et al., 2017)	NCT03381729	AAV9-SMN	Intrathecal	Phase I (2021)	AveXis, Inc.
X-Linked myotubular myopathy	NCT03199469	AAV8-hMTM1	Intravenous	Phase II (2025)	Audentes Therapeutics

### Parkinson’s Disease

Parkinson’s disease (PD) is neurodegenerative disorder caused by loss of dopaminergic neurons in the substantia nigra, leading to bradykinesia, rigidity, tremor, and gait dysfunction. Expressing neurotrophic factors such as glial cell-derived neurotrophic factor (GDNF) were tested in a single clinical trial and neurturin (NTN) was investigated in three clinical trials in PD patients. The rationale for delivering these neurotrophic factors was not to target a causative pathological molecular pathway but to provide neurotrophic support to the degenerating neuronal population. Although AAV delivery of these neurotrophic factors were well-tolerated in patients, the efficacies are unclear. However, these studies were important to demonstrate the feasibility and tolerability of intraparenchymal delivery of gene therapy directly in the human brain.

Most gene therapy approaches for PD aim to restore dopamine production in neurons. Delivery of 1-amino acid decarboxylase (AADC), a key enzyme for dopamine production, showed to be more efficacious when compared to neurotrophic factors. Proof of concept studies in NHP based on delivery of AADC by AAV showed an increase in levels of dopamine which was sustained for up 7 years (Bankiewicz et al., 2000, 2006). The first AAV delivered AADC study in humans (NCT00229736) was generally well-tolerated with some minor side effects (Eberling et al., 2008; Christine et al., 2009). A significant improvement of Parkinson’s Disease Rating Scale (UPDRS) score was reported at 6 months post-surgery and was sustained for up to 2 years. These promising results have triggered other trials based on AADC delivery and currently there are several clinical trials being conducted with higher doses and larger cohorts of patients. A clinical trial using a intrastriatal delivered lentivirus (ProSavin) expressing three key dopamine biosynthetic enzymes (tyrosine hydrolase, AADC, and GTP cyclohydrolase-1) for continuous dopamine production also demonstrated to be safe and well-tolerated with no surgical complications (CT01856439) (Palfi et al., 2014; Piguet et al., 2017). A significant improvement of motor function was observed, and a long-term analysis is ongoing. Another lentiviral gene therapy that is currently being tested in clinic and is very similar to ProSavin also expresses tyrosine hydroxylase, AADC AND GTP-cyclohydrolase (NCT03720418). While there is no data available yet from this study, the preclinical results in human primary neuronal cultures and NHP showed higher dopamine production compared to ProSavin (Badin et al., 2014).

### Alzheimer’s Disease

Alzheimer’s disease (AD) is the most common cause of age-related dementia affecting more than 40% of individuals of 85 years and older. More than 100 therapeutic compounds have been tested to date, but all failed to positively modify the course of the disease. Most gene therapy clinical trials for AD are based on intracerebral delivery of AAV-NGF. NGF encodes for nerve growth factor and is similar to GDNF and NTN in that it could provide neurotrophic support to neurons. Preclinical studies have shown that NGF can prevent degeneration of adult cholinergic neurons in the fore brain after injury (Kromer, 1987; Hefti, 2018). Although the surgical procedures and the treatments proved to be safe in humans, the efficacy of the studies were inconclusive. A more recent study for AD is based on intracisternal delivery of AAVrh.10hAPOE2 (NCT03634007). It was shown that inherence of an extra allele of *APOE4* gene, a variant of apolipoprotein E (APOE), significantly increases the risk for developing sporadic AD. However, *APOE2* homozygotes are protected against the disease and it is hypothesized that APOE2 delivery could be beneficial in APOE4 homozygous patients (Rosenberg et al., 2018). Whether this approach indeed modifies the course of the disease is yet to be demonstrated.

### Batten Disease

Batten disease or neuronal ceroid lipofuscinosis (NCL) is a rare group of neurodegenerative disorders that can manifest in infants, children, and adults. Symptoms include epilepsy, loss of cognitive and motor function, degeneration of the retina leading to blindness, and early death. More than a dozen CLN genes and more than 430 loss of function mutations have been linked to the disorders (Mole and Cotman, 2015). AAV-based clinical trials for Batten disease have mainly focused on delivery of AAV-CLN2 and AAV-CLN3. The first study (NCT00151216), using intracranial delivery of AAV2-CUhCLN2 showed little therapeutic benefit. A follow up study is still ongoing and used a different vector (AAVRh.10CUhCLN2) but abnormal T2 hyperintensities was observed in first dosed patients. Therefore, the dose was lowered for the following patients. For CLN3, one study (NCT03770572) is still recruiting and is based on AAV9-CLN3 delivery. For CLN6, a study with intrathecally delivered scAAV9.CB.CLN6 is ongoing. According to a press release, preliminary data from the first two dosed patients showed no further progression of the disease (according to the Hamburg motor and language score) during the first 2 years after dosing.

### Mucopolysaccharidoses (MPSs)

Mucopolysaccharidoses (MPSs) are rare metabolic diseases that are caused by impaired degradation of mucopolysaccharides, leading to accumulation of glycosaminoglycans in the lysosome. Symptoms can be detected as early as during the prenatal period but can also occur in adulthood. The symptoms in adults include difficulty of speech, ataxia, weakness, and dyskinesia, while children usually show neurological impairment, developmental delay, and premature death (Platt, 2018). Several gene therapy clinical trials are currently in progress for the treatment of MPS I (Hurler infantile syndrome or Hurler-Scheie and Scheie for the juvenile and adult forms), MPS II (Hunter syndrome), MPS IIIA and MPS IIIB (Sanfilippo syndromes A and B). For MPS I and MPS II, the trials are based on intravenous AAV delivery of ZFN therapeutic agents to insert a functional copy of alpha-L-Iduronidase (IDUA) or iduronate 2-sulfatase (IDS), respectively, into the albumin locus of patient hepatocytes. These trails are currently being tested in adult patients aiming to improve the peripheral symptoms of these diseases. For MPS IIIA, the main goal is to deliver a functional copy of the human *N*-sulfoglucosamine sulfohydrolase (h.SGSH) directly into the brain of young children. Thus far, h.SGSH delivery seems to be safe, but the therapeutic benefit was limited with cognitive improvement observed in only one patient (Vincent et al., 2014). For the treatment of MPS IIIB, the goal is to deliver *N*-sulfoglucosamine sulfohydrolase (NAGLU) to restore its expression in patients. Both intracerebral and intravenous delivery of AAV-NAGLU have shown to be promising in preclinical studies using rodent and dog models but whether this translates to therapeutic benefit in patients is yet to be demonstrated (Ellinwood et al., 2011; Fu et al., 2011).

### Pompe Disease

Pompe disease is a progressive neuromuscular disorder and is caused by a mutation in the acid alpha-glucosidase (GAA) gene, which encodes an enzyme required to degrade lysosomal glycogen (Corti et al., 2015). Accumulation of glycogen in multiple tissues results in cardiac, respiratory, and skeletal muscle dysfunction. Preclinical studies in GAA knockout mouse models showed rescue of glycogen accumulation in muscle and the central nervous system, and increased survival upon delivery of GAA by AAV (Puzzo et al., 2017). Delivery of GAA by AAV is currently being tested in three different clinical trials.

### Spinal Muscular Atrophy

Spinal muscular atrophy (SMA) is caused by the loss of function of the gene encoding the survival motor neuron 1 (SMN1) protein and is characterized by progressive loss of the lower motor neurons. The most severe form is type 1 SMA which affect infants. Patients usually die before the age of 20 months. Several clinical trials are being conducted by AveXis based on AAV9-SMN delivery. Thus far, remarkable clinical benefit was observed including increased motor functions, head control, sitting, rolling over, and speaking. The high dose of intravenous injected AAV9-SMN led to an increase in serum aminotransferase, a sign of liver toxicity, which was managed by oral prednisolone. Intrathecal delivery of AAV9-SMN is also being investigated by AveXis as improved transduction of the CNS was observed at lower doses as compared to intravenous administration in NHP. This program is currently also being investigated in clinic for other types of SMA (NCT03306277, NCT03505099, NCT03461289, and NCT03381729), results of which are pending (Table 4). A Biological License Application was submitted to the US FDA in November 2018 by Novartis, which acquired AveXis. In March 2019, Novartis announced the FDA approval of AAV9-SMS (Zolgensma®) for the treatment of pediatric SMA type 1 patients.

### Huntington’s Disease

Huntington’s disease (HD) is the most common autosomal dominant neurodegenerative disorder worldwide (Fisher and Hayden, 2014; Rawlins et al., 2016). The genetic cause is an expansion of 39 CAG triplets or more in first exon of the huntingtin (HTT) gene (Miniarikova et al., 2018). The mutated gene produces a mutant HTT protein that contains a long polyglutamine (polyQ) tract and results in a toxic gain of function. The mutant HTT protein causes neuropathology affecting the entire brain, with medium spiny neurons of the striatum being particularly vulnerable at early stages (Ross and Tabrizi, 2011; Bates et al., 2015). The clinical symptoms include progressive motor, cognitive, and psychiatric disturbances. Currently there is no treatment available that would halt or delay disease progression. Using artificial DNA or RNA molecules to achieve lowering of HTT translation as a potential therapy for HD has been broadly investigated in preclinical studies but few have made it to clinical trials. IONIS Pharmaceuticals was the first to initiate a Phase I/II clinical trial (NCT02519036) in 2015 based on HTT lowering with an intrathecally administered antisense oligonucleotide targeting both wild-type and mutant HTT. This trial was safe and a reduction of HTT protein was detected in the CSF (Tabrizi et al., 2019). Roche is continuing this study with a large phase III trial (NCT03761849) that was launched in 2018 and will be conducted at around 80–90 sites in about 15 countries. It remains unknown whether this intrathecally delivered ASO will reach the deeper brain structures to achieve sufficient lowering of the mutant HTT in the striatum. Concerningly, the latest published data using this approach showed a dose- and time-dependent increase of the ventricular volume, which suggests that the treatment caused more CNS atrophy, instead of the desired prevention of CNS atrophy (Tabrizi et al., 2019).

uniQure launched the first AAV-based RNAi therapy for HD in the second half of 2019 and the first two patients have been treated (ref. clinical trial: NCT04120493). The therapeutic candidates in AAV5-miHTT (AMT-130), which is anti-HTT miRNA targeting both wild-type and mutant HTT in a region close to the repeat in exon 1. AMT-130 was injected directly in the striatum of the brain and should provide a long-lasting production of miHTT. Due to the close proximity of miHTT target site to the CAG repeat, it is expected that AMT-130 can also target the short HTTexon1 mRNAs which are highly toxic. New preclinical data presented at the 15th Annual CHDI Huntington’s disease Therapeutics Conference demonstrated that Q175FDN mouse model, treated with AMT-130 experienced a dose-dependent lowering of exon 1 HTT mRNA in both the striatum and cortex.

## Safety and Delivery of Gene Therapy to Patients

The use of AAV vectors as transgene delivery has become one of the safest and most reliable methods to obtain sustained expression of a therapeutic transgene. Non-viral delivery methods using nanoparticles or ASOs can also deliver therapeutic agents but the need for recurrent injections can become overwhelming and increase the risk for infections in patients. Nevertheless, ASOs have shown to be efficient and safe in animal models and have led to the initiation of several clinical trials for SOD1-ALS, *C9orf72*-ALS, and SMN2 for SMA (Miller T. et al., 2013; Meyer et al., 2015; Mendell et al., 2017).

The delivery of gene therapy to the affected cells in patients suffering from neurodegenerative diseases is currently a major challenge. Several studies have implicated that the utility of AAV vectors for gene transfer is mainly determined by the capsid. We believe that this statement is partly true as the capsid is involved in several key cell entry steps. However, the route of administration is equally important as capsids behave differently upon different administration routes due to different microenvironments (Pietersz et al., 2020). For example, it was demonstrated that intraparenchymal injection of AAV5 resulted in high transduction of the rat brain (Pietersz et al., 2020). Similar findings were observed in a transgenic minipig model showing successful transduction at the injection site (striatum) and its surrounding area (Evers et al., 2018). In addition, both anterograde (e.g., to caudate) and retrograde (to cortex) AAV5 viral transport was observed, suggesting that the vector can travel along axons and transduce distant areas. While this route is promising for diseases with a more localized pathology, such as HD, it is unlikely to be sufficient in multifocal neurological pathologies such as ALS because anterograde and retrograde transport to cerebellum, brain stem, and spinal cord was not observed in this study. In Rats, intrathecal administration led to a more even distribution of the vector within the CNS and is potentially more relevant for diseases with multifocal neurological pathologies (Pietersz et al., 2020). However, it remains a challenge to deliver a therapeutically relevant dose throughout the entire CNS in larger animals. Another crucial aspect is safety, especially due to the irreversible nature of current gene therapies, evaluation of short term and long-term toxicity is critical. Other crucial parameters that needs to be addressed during preclinical studies are prediction of on- and off-target effects, immunogenicity, dose finding, timing of treatment, and the ability to modulate gene expression.

### Prediction of Safety, On- and Off-Target Effect

The most important aspect for drug development is safety and tolerability in patients. Both transgene products and delivery vector could lead to toxicity. Several clinical studies have demonstrated that administration of AAV in human CNS is safe, but immune responses should still be investigated during preclinical and clinical studies. The transgene products that are delivered could also induce on- and off-target effects, for example in the case of RNAi-based gene therapy. Prediction of these unwanted side effects can be evaluated early in preclinical studies using the currently available tools such as iPSC-technology, animal models, and bioinformatics to increase safety and tolerability in patients.

### Timing of Treatment

The success of a gene therapy is likely to be determined by the timing. Most of the adult onset neurodegenerative diseases have a pre-symptomatic stage and it may take several years to gradually progress into a severe state with progressive neuronal death. While neuronal death cannot be reversed, emerging evidence suggests that neurons in atrophic state can regenerate their normal functions (John and Petitto, 2014). Thus, early treatment before neuron death occurs is likely to provide better protection and could decelerate neuronal death. Currently all gene therapy clinical trials are performed during the manifest state in patients while treatment during pre-manifest could be more beneficial. With the current knowledge and technologies, neurodegenerative diseases with a family history can be diagnosed prenatally or later in life during the pre-symptomatic phase using genetic screening. The discovery and characterization of more clinically validated biomarkers that correlate with disease progression will also contribute to early diagnosis of both familial and sporadic neurodegenerative diseases. Biomarkers that track disease progression and correlate predictably in response to a therapeutic intervention can also greatly support future clinical trials by reducing the duration of the studies and number of patients that need to be followed. This is especially important in pre-manifest subjects when no clinical measures of disease progression can be applied. However, due to the lack of effective treatment options diagnoses during the premanifest stage also raises many ethical concerns and even patients at a high risk of developing these diseases are fearful to do a genetic screening. Patients who test positive are currently faced with difficult decisions that can have mayor psychological and financial impacts on the patients themselves and their families. For example, a positive genetic test could have implications on the daily life of a person, their health insurance, their ability to find a job, and on decision making with may affect their family (Cozaru et al., 2016; Uhlmann and Roberts, 2018). Ultimately, a positive genetic test may have consequences regarding a person’s ability to get a mortgage and integrate into society (Wauters and Van Hoyweghen, 2018). This is a phenomenon known as “*genetic discrimination*” and needs further attention. The success of new therapeutics will hopefully extend to trials in premanifest carriers and could make it easier for patients at risk to make the decision to do a genetic screening.

### Modulation of Gene Expression

Besides constitutively active promoters to drive transgene expression, the ability to “turn on” expression of a therapeutic molecule when it is needed and to “turn off” its expression in case of unwanted effects would also add considerably to the safety profile of any genetic therapy. The feasibility of different inducible transgene expression systems to use in combination with gene therapy has been demonstrated in animal studies but translation to the clinic is lacking (Cheng et al., 2018; Liefhebber et al., 2019). The incorporation of the inducible systems to regulate gene therapy would add to the safety by making adjustments on individual basis possible and preventing too high concentrations of transgene products that could be detrimental. However, there is still room for improvement to make inducible systems suitable for clinical applications. One drawback is the basal activity of these systems in the “off-state.” Especially small transgenes such as non-coding RNAs tend to be leaky when using inducible systems and further optimizations are needed. Another aspect that needs more attention isobtaining of sufficient transgene expression once in the on-state. Especially for CNS diseases, the inducer drug needs to efficiently cross the BBB and reach the target tissue at concentrations that are high enough for therapeutically relevant expression of the transgene. Possible immune responses to elements inherent to the currently available inducible systems and side effects of the inducer drug should also be considered for clinical development. The co-development of two different products (inducible system components and transgene) in either a single or duo vector also add more parameters to the safety concerns of gene therapies, but these systems also offer notable advantages. Being able to modulate transgene expression will at least reduce safety concerns of the permanency expressed transgene. In turn making further investigation and optimization of these systems highly attractive for application in gene therapy.

### Future Perspectives

Gene therapy holds great promise for delivering therapeutic genes to treat neurological disorders. AAV vectors are currently considered one of the safest vehicles to treat CNS disorders. Several serotypes are available and as the potential of gene therapy is increasingly recognized, there is emerging need for new AAV serotypes. There is an enormous desire for new AAV vectors with improved transduction profile, better distribution, and higher transduction in the target organs upon less invasive administration routes. The genetic modification of AAV vectors and engineering AAV vectors could overcome these hurdles. The selection of appropriate delivery routes is also an important factor for the success of gene therapy. Currently, direct administration of AAV vectors into the parenchyma is preferred for efficient transduction of the CNS. Although this route of administration is invasive, the advantages over injections into the venous system or other fluid-filled compartments are clear. Intra-parenchymal administration provides high concentration of the transgene in the target cells, high local transduction, less distribution to other organs, and lower risk for immune responses or toxicities due to AAV particles or ectopic expression of the transgene. Administration of AAV via systemic or intrathecal routes require higher doses which in turn increase the risk for toxicity. There is also a lot of room for improvement at the transgene level. For example, generation of new promoters and *cis-*elements, optimization of codons, and more efficient transgene design could improve efficacy and restrict expression in specific CNS cell types.

Throughout this review several clinical trials on AAV-based gene therapies for CNS disorders have been discussed (Piguet et al., 2017). Although all studies showed that AAV is safe and tolerated well in the human CNS, few of these studies have been efficacious in demonstrating therapeutic benefit. Too low transduction of the target organs could have played an important role, but one major problem is the lack of good predictive preclinical models for a more accurately translation of favorable preclinical outcomes to the clinic. Many regions of the CNS are difficult to access after *in vivo* delivery of viral vectors, resulting in a reduced number of cells expressing the therapeutic transgene which may be insufficient to reach overall required therapeutic levels (Piguet et al., 2017). Furthermore, the discovery of biomarkers for early detection of the diseases will be important to identify new patients and to better predict clinical outcome. More importantly, delivering therapies prior to onset of neurodegeneration will be key to improving efficacy of gene therapies for neurodegenerative diseases. A better understanding of factors leading to these diseases will also facilitate development of better pre-clinical models that make it possible to better predict clinical success. Results from clinical trials are raising and the successes and lessons learned from the past and current AAV gene therapy clinical trials will be highly valuable for application to a wider range of neurodegenerative and neuromuscular diseases.

## Author Contributions

Both authors listed have made a substantial, direct and intellectual contribution to the work, and approved it for publication.

## Conflict of Interest

RM and PK were employed by the uniQure Biopharma B.V.

## Footnotes

1 www.mirbase.org
